# Supplementary material for: Microscale chemical imaging to characterize and quantify corrosion processes at the metal-electrolyte interface
Source: Npj Mater Degrad. 2024 Nov 6;8(1):116. doi: 10.1038/s41529-024-00534-x (PMC11540903; doi:10.1038/s41529-024-00534-x)
Supplement: Supplementary file 1 — Supplementary information [file 41529_2024_534_MOESM1_ESM.pdf]

Microscale chemical imaging to characterize and quantify corrosion processes at the metal-electrolyte interface

Cristhiana C. Albert[1], Shishir Mundra[1], Dario Ferreira Sanchez[2], Fabio E. Furcas[1], Ashish D. Rajyaguru[2], O. Burkan Isgor[3], Daniel Grolimund[2], Ueli M. Angst[1,*]

[1]Institute for Building Materials, ETH Zurich, Zurich, Switzerland

[2]Paul Scherrer Institute, Villigen, Switzerland

[3]School of Civil and Construction Engineering, Oregon State University, USA

*corresponding author

Supplementary Information

1. Supplementary Note 1 - Evolution of corrosion processes over time

Replicate capillaries containing an iron-electrolyte interface presented specific patterns of solid phases precipitation, containing regions with high amounts of corrosion products separated by gap regions with negligible precipitation. The precipitation started in regions further away from the metal, and over time it developed into areas closer to the metal-electrolyte interface (Supplementary Figure 1a). The colours of the corrosion products reproducibly changed from a light-yellow to brown-orange, particularly closer to the metal surface (Supplementary Figure 1b). In the case of Supplementary Figure 1c, both sides of the capillary remained open during the experiment, promoting O_2 access to the electrolyte and the precipitation of corrosion products. This supports that corrosion products only formed in the presence of O_2 in the iron-electrolyte case and time frame studied here.

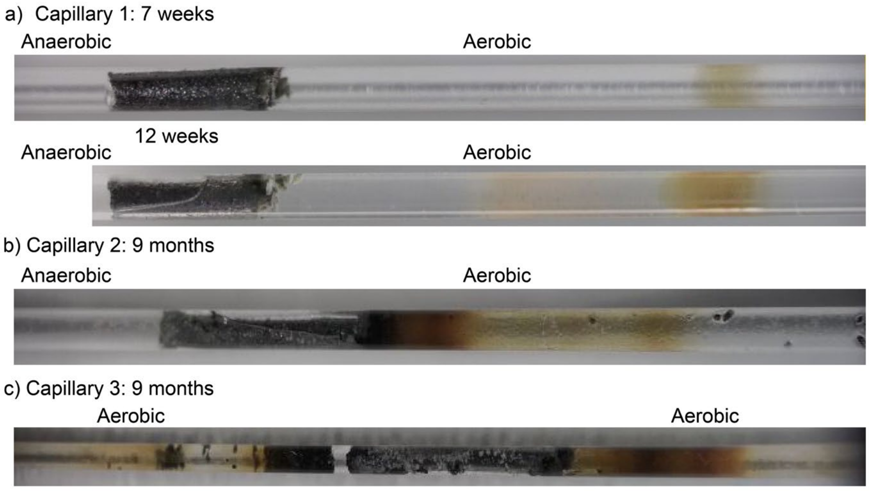

Supplementary Figure 1 – Examples of replicate capillaries containing two metal-electrolyte interfaces and corrosion products precipitating in specific patterns in the aerobic side of capillary: a) capillary 1, microscopy of corrosion products at 7 and 12 weeks; b) capillary 2, microscopy of corrosion products at 9 months; and c) capillary 3, microscopy of corrosion products at 9 months, with two aerobic sides.

2. Supplementary Note 2 - Phase characterization of corrosion products

2.1. X-ray diffraction analysis

The structure of the solid phases in the capillary was assessed by μX-ray diffraction (μXRD) mapping. The corrosion products were identified as goethite and 6L-ferrihydrite, as exemplified in Supplementary Figure 2b-c. Region I presented typical scattering patterns of the amorphous electrolyte (point A, Supplementary Figure 2d), while region II had more crystalline phases, with peaks of goethite and 6L-ferrihydrite (points B, C in Supplementary Figure 2e,f). In comparison, region III exhibited a significant background of the amorphous electrolyte, still containing some crystalline peaks in points D, E, F (Supplementary Figure 2g-i), while point G (Supplementary Figure 2j) presented less significant peaks.

To facilitate the identification of peaks in regions IV, V and VI, the XRD patterns were treated by subtracting the average amorphous halo obtained from the regions of the capillary only containing the electrolyte and negligible iron counts. Therefore, the mostly flat XRD patterns in these regions are associated with the electrolyte (e.g., point I, Supplementary Figure 2l). Broader peaks were also found in these XRD patterns and are indicative of nanocrystalline phases, found in region V (points J, K, Supplementary Figure 2m,n) and in hot spots along regions IV and VI of the capillary (points H, L, Supplementary Figure 2k,o).

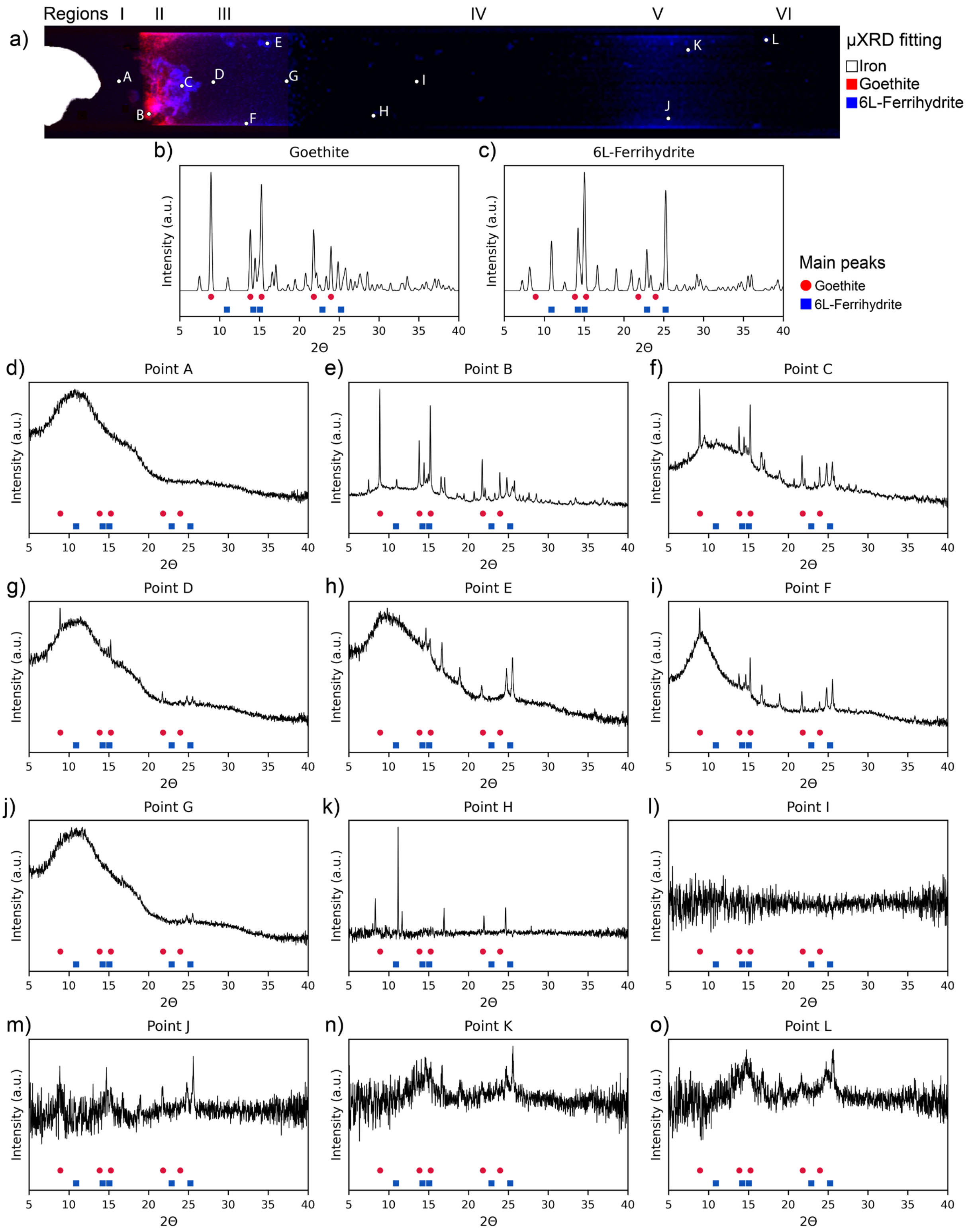

Supplementary Figure 2 – Overview of XRD patterns along the capillary, with (a) µXRD mapping identifying the distribution of iron (white), goethite (red) and 6L-ferrihydrite (blue), and locating examples of points A-L with XRD patterns indicated in (d)-(o), and the XRD patterns of the standards (b) goethite and (c) 6L-ferrihydrite.

2.2. XANES analysis

X-ray absorption near edge structure (XANES) at the iron K-edge was performed in selected spots on the aerobic side of the capillary. The XANES region contains the pre-edge feature, associated with the electron transition 1s → 3d [1–3]. The pre-edge provides information on the oxidation state and coordination environment of the iron in the corrosion products, independent of the crystallinity of the phases.

The μXRD mapping identified goethite and ferrihydrite as the corrosion products. With XANES, these phases can be differentiated by the coordination environment of iron, associated with the shape of the pre-edge feature, as observed in Supplementary Figure 3 [2,3]. Goethite has an octahedrally coordinated iron and a low intensity split pre-edge feature [2]. In this centrosymmetric iron phase, the only mechanism behind the 1s → 3d transition is the allowed electric quadrupole transition, which produces two excited states with a distinguishable energy split between them. In contrast, ferrihydrite has a tetrahedrally coordinated iron, and its pre-edge feature appears as a high intensity single peak [2]. In this case, the electronic 1s → 3d transition happens both through the allowed electric quadrupole transition and the electric dipole mechanism associated with 4p mixing into 3d orbitals. The resultant excited states have a non-resolvable energy shift at the pre-edge, whose merged contribution leads to an apparent single peak. Additionally, we identified a third flat peak at higher energies in the pre-edge of ferrihydrite, which has been associated with 3d orbitals from distant iron neighbours [3–5].

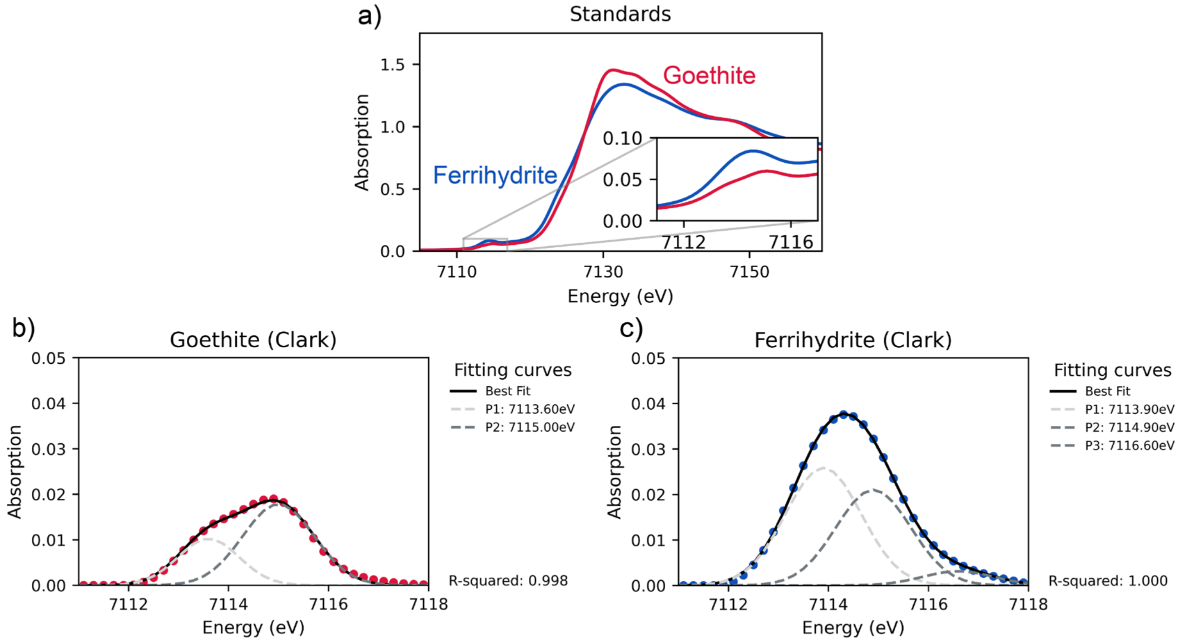

Supplementary Figure 3 – Overview of XANES spectra of standards, showing (a) the complete XANES region of goethite and ferrihydrite [6], and the details of pre-edge peak fitting of (b) goethite and (c) 6L-ferrihydrite.

The contribution of goethite and ferrihydrite in the measured spots of the capillary (Supplementary Figure 4a) was determined with linear combination fitting of their pre-edges, and some typical examples are discussed. In region I, point 1 did not exhibit a well-formed feature resembling an iron pre-edge (Supplementary Figure 4b), consistent with its position in the electrolyte region not exhibiting iron phases, and thus it was not used for the fitting; differently, point 2 showed an iron pre-edge feature mostly fitted as ferrihydrite (Supplementary Figure 4c,d). In region II, with the highest amount of corrosion products, point 4 had a lower

intensity and partially split pre-edge peak, indicating a higher contribution of goethite (Supplementary Figure 4e,f). In comparison, points 8 and 13 presented a single-peak shape, more alike ferrihydrite (Supplementary Figure 4g-j), showing the decreased goethite content in the less concentrated zones of the capillary. The hot spot 14 in region IV showed a mix of ferrihydrite and goethite (Supplementary Figure 4k,l). Another increase in the goethite content was found in region V, as represented in point 19 (Supplementary Figure 4m,n), followed by its decrease further away in point 21 of region VI (Supplementary Figure 4o,p).

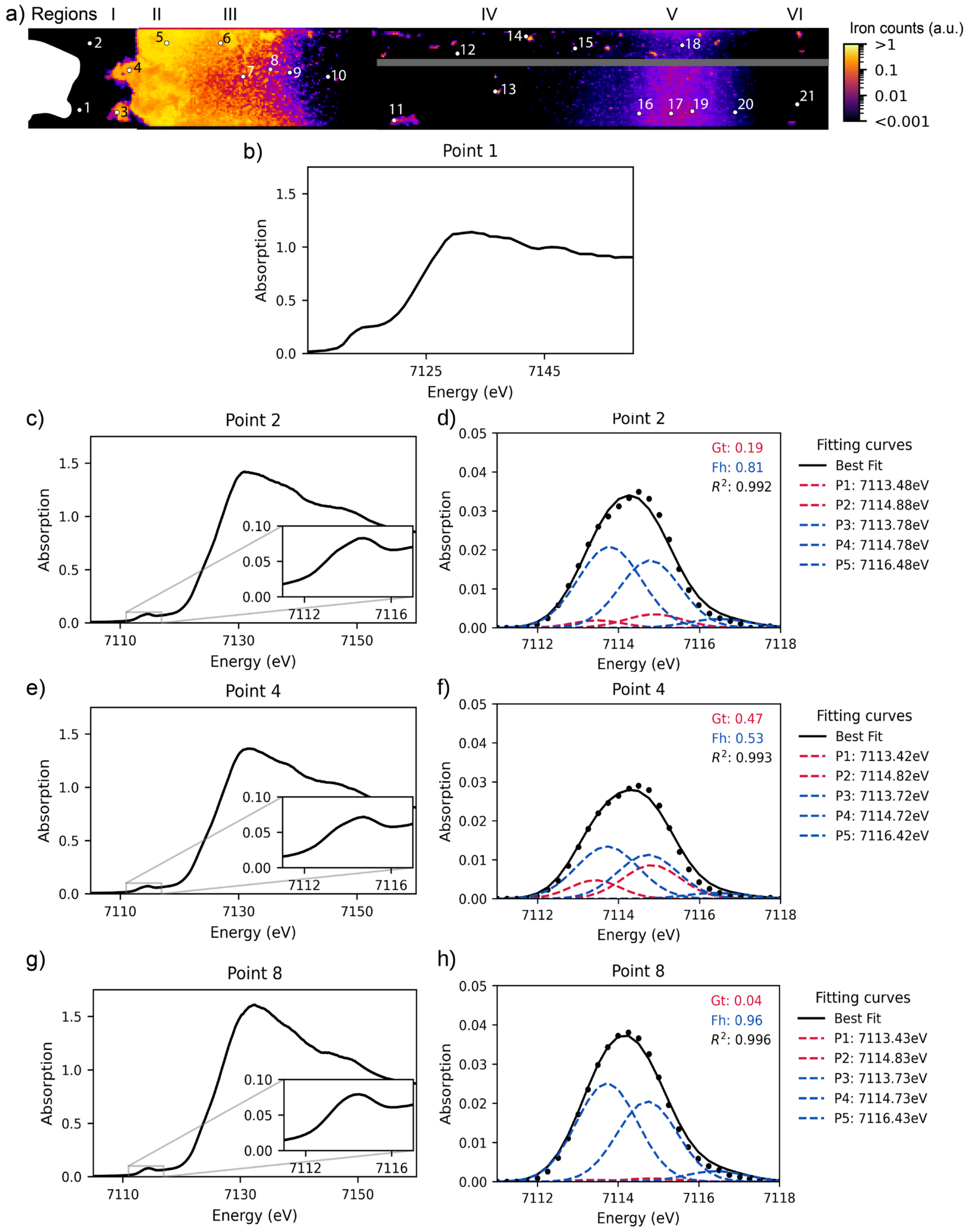

Supplementary Figure 4 – Overview of XANES spectra and pre-edge fitting, with (a) µXRF mapping showing the selected spots where XANES was conducted, and (b)-(h) XANES spectra of selected points, with details of pre-edge peak fitting (part 1).

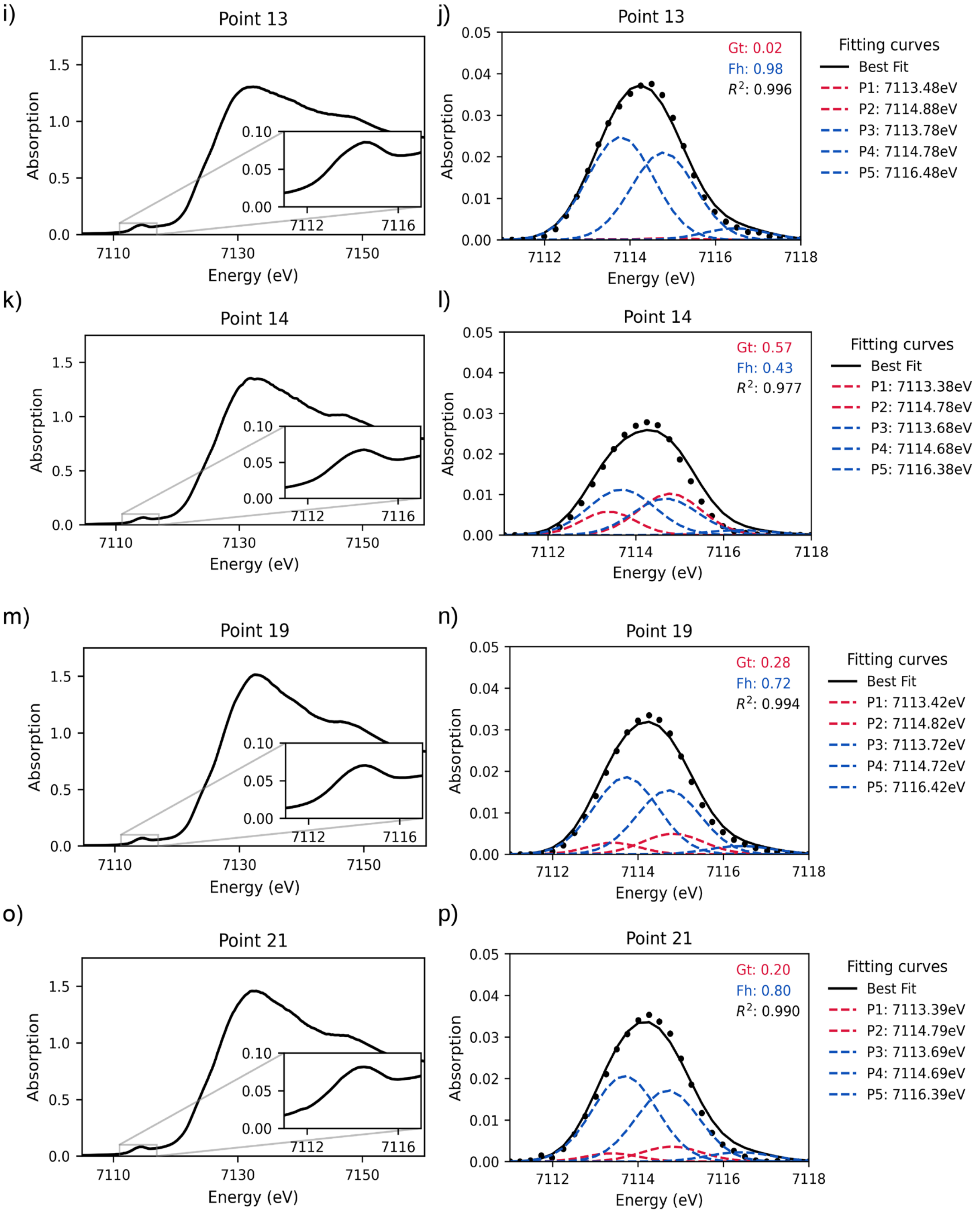

Supplementary Figure 4 – Overview of XANES spectra and pre-edge fitting, including (i)-(p) XANES spectra of selected points, with details of pre-edge peak fitting (part 2).

3. Supplementary Note 3 - µX-ray transmission data

The µX-ray transmission measurements at 19.1 keV yielded the µX-ray attenuation contrast mapping of the capillary (Supplementary Figure 5a). The contribution of the capillary glass walls was removed (Supplementary Figure 5b), and the remaining attenuation was attributed to the corrosion products and employed on their quantification, according to section 4.2.5 of the paper. As emphasized there, a projection artefact of the capillary walls appears on the top and bottom parts of the image, leading to a local overestimation of the corrosion products in up to 15%.

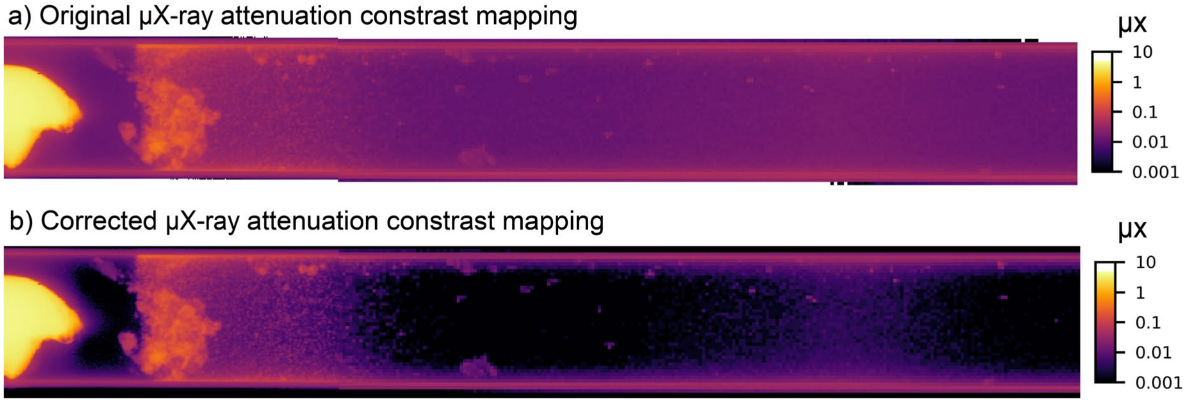

Supplementary Figure 5 – Transmission data overview, with a) original and b) corrected µX-ray attenuation contrast mapping of the capillary, obtained through the transmission measurements.

4. Supplementary Note 4 – Chemical and electrochemical reactions

The reactions associated with the electrochemical cell in the capillary setup are listed below. The anodic reaction corresponds to the iron dissolution, while the possible cathodic reactions are O_2 reduction in aerobic conditions, H_2 evolution and HCO_3^- reduction.

Fe dissolution $Fe \rightarrow Fe^{2+} + 2e^-$ (anodic reaction)

O_2 reduction $\frac{1}{2}O_2 + H_2O + 2e^- \rightarrow 2OH^-$ (main cathodic reaction at pH 8)

H_2 evolution $2H_2O + 2e^- \rightarrow H_2 + 2OH^-$ (secondary cathodic reaction)

HCO_3^- reduction $2HCO_3^- + 2e^- \rightarrow + H_2 + CO_3^{2-}$ (secondary cathodic reaction)

The reactions associated with the precipitation of corrosion products in the capillary setup are listed below, following the oxidation of Fe(II), precipitation of ferrihydrite and transformation to goethite by a precipitation-redissolution process [7–11]:

Fe(II) oxidation $Fe^{2+} \rightarrow Fe^{3+} + e^-$ (accelerated by O_2)

Ferrihydrite precipitation $Fe^{3+} + 3H_2O \rightarrow Fe(OH)_3(s) + 3H^+$

Ferrihydrite redissolution $Fe(OH)_3(s) \rightarrow Fe(OH)_3(aq.)$

Fe(III) release $Fe(OH)_3(aq.) + 3H^+ \rightarrow Fe^{3+} + 3H_2O$

Goethite precipitation $Fe^{3+} + 2H_2O \rightarrow \alpha\text{-}FeOOH(s) + 3H^+$

References

[1] G.A. Waychunas, M.J. Apted, G.E. Brown, X-ray K-edge absorption spectra of Fe minerals and model compounds: Near-edge structure, Phys Chem Minerals 10 (1983) 1–9. https://doi.org/10.1007/BF01204319.

[2] T.E. Westre, P. Kennepohl, J.G. DeWitt, B. Hedman, K.O. Hodgson, E.I. Solomon, A Multiplet Analysis of Fe K-Edge 1s f 3d Pre-Edge Features of Iron Complexes, S f (n.d.).

[3] M. Wilke, F. Farges, P.-E. Petit, G.E. Brown, F. Martin, Oxidation state and coordination of Fe in minerals: An Fe *K*- XANES spectroscopic study, American Mineralogist 86 (2001) 714–730. https://doi.org/10.2138/am-2001-5-612.

[4] C. Mikutta, X-ray absorption spectroscopy study on the effect of hydroxybenzoic acids on the formation and structure of ferrihydrite, Geochimica et Cosmochimica Acta 75 (2011) 5122–5139. https://doi.org/10.1016/j.gca.2011.06.002.

[5] G. Dräger, R. Frahm, G. Materlik, O. Brümmer, On the Multipole Character of the X-Ray Transitions in the Pre-Edge Structure of Fe K Absorption Spectra. An Experimental Study, Physica Status Solidi (b) 146 (1988) 287–294. https://doi.org/10.1002/pssb.2221460130.

[6] Clark, A.H., XANES spectra of goethite and ferrihydrite measured at SuperXAS beamline of PSI-SLS (Personal communication), (2024).

[7] F.E. Furcas, B. Lothenbach, S. Mundra, C.N. Borca, C.C. Albert, O.B. Isgor, T. Huthwelker, U.M. Angst, Transformation of 2-Line Ferrihydrite to Goethite at Alkaline pH, Environmental Science and Technology 57 (2023) 16097–16108. https://doi.org/10.1021/acs.est.3c05260.

[8] U. Schwertmann, H. Stanjek, H.-H. Becher, Long-term *in vitro* transformation of 2-line ferrihydrite to goethite/hematite at 4, 10, 15 and 25°C, Clay Miner. 39 (2004) 433–438. https://doi.org/10.1180/0009855043940145.

[9] S. Das, M.J. Hendry, J. Essilfie-Dughan, Transformation of Two-Line Ferrihydrite to Goethite and Hematite as a Function of pH and Temperature, Environ. Sci. Technol. 45 (2011) 268–275. https://doi.org/10.1021/es101903y.

[10] S.A. Chen, P.J. Heaney, J.E. Post, P.J. Eng, J.E. Stubbs, Hematite-goethite ratios at pH 2–13 and 25–170 °C: A time-resolved synchrotron X-ray diffraction study, Chemical Geology 606 (2022) 120995. https://doi.org/10.1016/j.chemgeo.2022.120995.

[11] U. Schwertmann, Effect of pH on the Formation of Goethite and Hematite from Ferrihydrite, Clays and Clay Minerals 31 (1983) 277–284. https://doi.org/10.1346/CCMN.1983.0310405.
